# Overaccumulation of miR-483-3p exerts acute toxic effects on ovarian granulosa cells by impairing cell proliferation, mitochondrial function, and METTL3-mediated m6A modification

**DOI:** 10.7717/peerj.21567

**Published:** 2026-07-27

**Authors:** Kaiyuan Shen, Xiaoli Dai, Liqun Chen, Yuanyuan Liang, Ping Huang, Yuxin Zeng, Xiaoli Qu

**Affiliations:** 1 Reproductive Medicine, Liuzhou People’s Hospital Affiliated to Guangxi Medical University, Liuzhou, Guangxi, China; 2 Liuzhou Key Laboratory of Reproductive and Genetic Metabolic Diseases, Liuzhou, Guangxi, China; 3 Research Service Office, Liuzhou People’s Hospital Affiliated to Guangxi Medical University, Liuzhou, Guangxi, China

**Keywords:** Diminished ovarian reserve, MiR-483-3p, Methyltransferase like 3 (METTL3), N6-methyladenosine (m6A), Cell proliferation, Mitochondrial membrane potential

## Abstract

**Background:**

Diminished ovarian reserve (DOR) is a common cause of female infertility. Prior high-throughput sequencing showed specific follicular fluid exosomal miRNA profiles in DOR patients, implicating exosomal miRNAs in DOR pathogenesis. Using miRNA PCR arrays, we found miR-483-3p was significantly upregulated in DOR follicular fluid exosomes. Bioinformatics and experiments indicated METTL3 as a potential miR-483-3p target, suggesting excess miR-483-3p exerts cytotoxicity and suppresses METTL3-mediated N6-methyladenosine (m6A) modification. This study thus investigated miR-483-3p effects on METTL3 expression, m6A levels, cell proliferation, and mitochondrial function in ovarian granulosa cells (GCs).

**Methods:**

The targeting interaction between miR-483-3p and METTL3 was confirmed using a dual-luciferase reporter gene assay. qRT-PCR, immunofluorescence, Western blot, and Dot blot were used to evaluate the effects of miR-483-3p mimics, cyclophosphamide (CTX), and STM2457 on METTL3 protein expression and m6A modification levels in KGN cells. Additionally, Cell Counting Kit-8 (CCK-8) and 5-Ethynyl-2’-deoxyuridine (EdU) assays, along with mitochondrial membrane potential (MMP) measurements, were used to examine the impact of these treatments on KGN cell proliferation and mitochondrial function.

**Results:**

miR-483-3p directly targeted the 3′ untranslated region (3′UTR) of METTL3, and its mimics significantly suppressed METTL3 gene expression. In contrast, CTX robustly promoted METTL3 gene expression, accompanied by a substantial elevation in global m6A levels. STM2457 treatment also showed a similar trend. But miR-483-3p overexpression modestly inhibited METTL3 protein and m6A levels. Regarding cellular phenotypes, miR-483-3p overexpression, CTX, and STM2457 all exerted significant inhibitory effects on KGN cells. All three treatments consistently suppressed the cell viability of KGN cells, reduced the EdU-positive cell ratio, and decreased MMP levels.

**Conclusion:**

This study provides preliminary evidence suggesting a specific regulatory interaction between miR-483-3p and METTL3. We demonstrate that aberrantly high expression of miR-483-3p significantly suppresses METTL3 mRNA levels but only moderately reduces METTL3 protein and m6A levels, suggesting the involvement of complex post-transcriptional regulatory mechanisms. Furthermore, overaccumulation of miR-483-3p markedly inhibits the proliferation of ovarian GCs and impairs their mitochondrial function. Further investigation into the role of the candidate miR-483-3p-METTL3-m6A axis in DOR will be conducted using *in vivo* models alongside an expanded cohort of clinical samples.

## Introduction

Diminished ovarian reserve (DOR) is strongly linked to declining female fertility and constitutes a critical determinant influencing the outcomes of assisted reproductive technologies (Zhu et al., 2023). This pathological condition may result from the interplay of multiple factors, including aging, genetic predisposition, and iatrogenic interventions (Han et al., 2022). However, its precise molecular regulatory mechanisms underlying this condition remain incompletely elucidated. Studies have demonstrated that exosomes within the follicular microenvironment regulate cellular activities by transporting bioactive molecules, particularly nucleic acids like microRNAs (miRNAs) (Di Pietro, 2016; Santonocito et al., 2014). These miRNAs exert a crucial regulatory influence in reproductive biological processes (Esfandyari et al., 2021; Zhao et al., 2022). These discoveries offer a novel research perspective for elucidating the pathogenesis and progression of DOR.

In our previous study, we employed high-throughput sequencing technology to identify a specific exosomal miRNA expression profile in the follicular fluid of DOR patients (Shen et al., 2023). Recently, we employed and validated the miRNA PCR array comprising miRNAs previously validated in the literature, derived from follicular fluid exosomes. By integrating the results of the miRNA PCR assay with those of the prior sequencing analysis, we identified that exosomal miR-483-3p was significantly upregulated. A previous study reported that miR-483-3p is abnormally elevated in certain malignant tumors (Kim et al., 2025). Notably, bioinformatics analysis revealed that the predicted target genes of miR-483-3p include several important methyltransferases, such as METTL3, METTL6, and METTL8. miR-483-3p specifically targets and inhibits the expression of METTL3 (Cheng et al., 2021), suggesting a role in modulating N6-methyladenosine (m6A) RNA methylation and cellular functions. Given that ovarian granulosa cells (GCs) are the primary cells responsible for maintaining ovarian function (Liu et al., 2023), we hypothesized that the overaccumulation of miR-483-3p may impair METTL3-mediated m6A modification and other cellular functions in GCs.

To validate this hypothesis, this study employed the human ovarian granulosa cell line KGN as an *in vitro* model. Specifically, the study implemented the following strategies to elucidate the underlying mechanisms: (1) transient transfection for miR-483-3p overexpression; (2) exposure to cyclophosphamide (CTX), a chemotherapeutic agent used to induce ovarian granulosa cell injury (Chen et al., 2024b); and (3) treatment with STM2457, a specific inhibitor of METTL3 methyltransferase activity (Di, Li & Li, 2025). We examined the impact of these interventions on METTL3 expression, m6A modification levels, cell proliferation, and mitochondrial function.

Therefore, this study aims to elucidate whether the overaccumulation of miR-483-3p exerts detrimental effects on GCs. We found that overexpression of miR-483-3p inhibited the METTL3 gene expression, and impaired the proliferative capacity and mitochondrial function of GCs. The results of this experiment will facilitate the exploration of the mechanisms by which miRNAs contained in follicular fluid exosomes regulate ovarian function. They will also aid in the development of treatment strategies aimed at restoring ovarian function.

## Materials & Methods

### Experimental group design

The experiment was designed with five groups as follows: (1) control group, with no treatment; (2) CTX group, treated with 500 µM CTX; (3) STM2457 group, treated with 12.5 µM STM2457; (4) miR-483-3p mimics group, transfected with 100 nM miR-483-3p mimics; and (5) mimics negative control (NC) group, transfected with 100 nM mimics NC. Details of the selection for the optimal working concentrations of CTX and STM2457 are shown in Fig. S1.

### Cell culture

The human ovarian granulosa cell line (KGN; Procell, SN: 7CJKMQMQQ3) was utilized as the *in vitro* model in this study. Cells were cultured in DMEM/F12 medium supplemented with 10% fetal bovine serum (FBS), 100 U/mL penicillin, and 100 µg/mL streptomycin. At approximately 90% confluence, the cells were detached using 0.25% trypsin-EDTA solution, passaged at a 1:2 split ratio, and then maintained in an incubator at 37 °C with 5% CO_2_.

### Total miRNA, RNA extraction, and qRT-PCR

Total RNA, including miRNA, was extracted according to the manufacturer’s instructions (CW0627S; Cwbio, Beijing, China). cDNA synthesis was performed using the miRNA First Strand cDNA Synthesis (Tailing Reaction) kit (B532451; Shanghai Sangon, Shanghai, China) for miRNA or the Thermo Scientific RevertAid Master Mix (M16325; Thermo Fisher Scientific, Waltham, MA, USA) for mRNA. Following synthesis, cDNA was stored at −20 °C and diluted by factors ranging from 10- to 1,000-fold for further use. qRT-PCR reactions of miRNA or mRNA were prepared according to the respective manufacturers’ instructions for the SYBR Green PCR kits (B532461; ABI, A25742; Shanghai Sangon, Shanghai, China) using the LightCycler^®^ 480 Instrument II (Roche, Basel, Switzerland). For miRNA reverse transcription, we employed a poly(A)-tailing approach; consequently, miRNA detection was performed using specific forward primers and a universal reverse primer. We used U6 and H2A.z as reference genes for miRNA and mRNA normalization in qRT-PCR, respectively. H2A.z is not a conventional reference gene, but our experiments showed that it is stably expressed in KGN cells, with quantification cycle (Cq) values consistently ranging from 18 to 22 (details are provided in the Data S1). Gene expression analysis was performed using the comparative threshold cycle (2^−ΔΔCt^) method. The primer sequences used in this experiment are detailed in Table 1. Each experimental group included at least three biological replicates and three technical replicates.

**Table 1:** Primer sequences.

Gene name	Sequence (5′→ 3′)
miR-483-3p	Forward: GTCGATCACTCCTCTCCTCCCCCGTC
*U* 6^*^	Forward: CTCGCTTCGGCAGCACA
	Universal Reverse: AGTGCAGGGTCCGAGGTATT
*METTL3*	Forward: GTTAGTCTCTGGTCTGAA
	Reverse: CTTGGCTGTTGTAGTATT
H2A.z^*^	Forward: GGCGCAAAGAGAAGCTGAAG
	Reverse: TCCTGGCTGTTCTTGTCCTC

**Notes.**

* Reference gene.

### miRNA PCR array

This study has been approved by the Ethics Committee of Liuzhou People’s Hospital (Approval No. KY2023-158-01). Before collecting follicular fluid samples, all patients had signed (or provided) fully informed consent. Follicular fluid samples were collected from three patients with DOR and three patients with normal ovarian reserve (NOR), all of whom were undergoing assisted reproductive technology (ART) (the patient characteristics are presented in Table 2). DOR was diagnosed based on the well-accepted international diagnostic criteria (Pastore et al., 2018). Briefly, a diagnosis of DOR required the presence of at least two of the following criteria: (i) AFC ≤ 5–7 on days 2–3 of menstruation, (ii) anti-Müllerian hormone (AMH) <0.5–1.1 ng/ml, and (iii) basal FSH >10 IU/L. Among these, AMH levels served as a key inclusion criterion for this study. The exosome extraction method was adapted from our previous research (Shen et al., 2023). Follicular fluid exosomal miRNAs were profiled using a human follicular fluid exosomal miRNA PCR array (WCgene, wc-miRNA0043-H). The PCR array plate includes 90 specific miRNA primers, four internal reference genes, and two blank controls (Data S1). Prior to loading, the PCR plate was centrifuged at 2,000 rpm for 20 s at room temperature. For each plate, the reaction mixture was prepared, consisting of 510 µL miRNA PCR mix, 100 µL cDNA, 290 µL nuclease-free water, and 20 µL ROX dye. Subsequently, 9 µL of the mixture followed by 1 µL of template was loaded into each well. The plate was subsequently sealed with a transparent film and centrifuged again at room temperature, 2,000 rpm, for 20 s. PCR was performed under the following conditions: pre-denaturation at 95 °C for 30 s (one cycle), followed by 40 cycles of amplification at 95 °C for 5 s and 60 °C for 30 s. Each biological sample group, consisting of technical replicates, was analyzed in triplicate. After PCR, data analysis was performed as follows: miRNAs with no detectable signals in all samples were excluded. The fold-change (FC) in expression was calculated. Differentially expressed miRNAs were identified using a threshold of —log_2_FC— > 1.5, and statistical significance was set at *P* < 0.05.

**Table 2:** Clinical characteristics of DOR and NOR patients.

Parameters	DOR (*n* = 3)	Normal (*n* = 3)	t	*P*
Age	36.33 ± 3.79	29.67 ± 2.08	2.673	0.056
BMI (kg/m^2^)	22.84 ± 2.08	25.77 ± 5.64	−0.845	0.446
Basal serum FSH (IU/L)	8.37 ± 1.99	5.66 ± 0.16	2.361	0.140
Basal serum LH (IU/L)	5.46 ± 1.00	4.88 ± 1.20	0.637	0.559
Basal serum PRL (ng/mL)	20.99 ± 8.48	16.50 ± 8.31	0.665	0.548
Basal serum E_2_ (pmol/L)	175.72 ± 80.58	229.10 ± 156.18	−0.526	0.627
Basal serum T (nmol/L)	0.55 ± 0.40	1.15 ± 0.43	−1.759	0.153
Basal serum P (ng/mL)	0.75 ± 0.04	0.91 ± 0.36	−0.780	0.516
AMH (ng/mL)	0.76 ± 0.29	3.90 ± 2.97	−1.819	0.208
AFC	7.67 ± 0.58	16.00 ± 7.00	−2.055	0.175

**Notes.**

All results are presented as the mean ± SD.

Abbreviations DOR diminished ovarian reserve NOR normal ovarian reserve BMI body mass index FSH follicle stimulating hormone LH luteinizing hormone PRL prolactin E2 estradiol T testosterone P progesterone AMH anti-Müllerian hormone AFC antral follicle count

### Target gene prediction and enrichment analysis

The prediction of miR-483-3p target genes was conducted using the databases miRanda (http://www.microrna.org/microrna/home.do), TargetScan (http://www.targetscan.org/), and miRbase (https://mirbase.org/). Gene Ontology (GO) and the Kyoto Encyclopedia of Genes and Genomes (KEGG) analyses were carried out using the DAVID database (https://davidbioinformatics.nih.gov/). The cut-off values were defined as the ratio of clustered genes to total genes ≥ 4% and *P* < 0.05, respectively.

### Protein-protein interaction network construction

PPI network construction and module analysis were conducted to explore the functional relationships of target genes. The PPI network was constructed using the Search Tool for the Retrieval of Interacting Genes (STRING) database (version 12.0; http://string-db.org).

### Dual-Luciferase reporter gene assay

Potential binding sites between miR-483-3p and METTL3 were predicted based on the ENCORI (https://rnasysu.com/encori) and miRanda (http://www.microrna.org/microrna/home.do) databases. To validate these predictions, METTL3 3′ untranslated region (3′ UTR) wild-type (WT) and mutant (MUT) fragments containing the predicted binding sites were cloned into the multiple cloning site (MCS) of the pmiR-RB-Report™ vector. The WT and MUT recombinant plasmids were transfected into 293T cells along with miR-483-3p miRNA mimics or mimics NC using Lipofectamine 3000 (L3000015; Invitrogen, Carlsbad, CA, USA). Renilla luciferase activity served as the internal control. After 48 h, the ratio of firefly to renilla luciferase activities was measured using the Dual-Luciferase Reporter Gene Assay Kit (KGAF040; Keygen, Vancouver, Canada). Each experimental group included three biological replicates.

### Cell transfection and drug treatment

KGN cells were seeded at densities of 5.0  × 10^5^ cells/well in 6-well plates and 2.0  × 10^3^ cells/well in 96-well plates, respectively. Cells were cultured overnight to reach 50%–70% confluence, an optimal density for subsequent transfection or drug treatment with CTX or STM2457. For transfection experiments, miR-483-3p mimics and mimics NC were transfected into cells using Lipofectamine 3000 according to the manufacturer’s protocol. For drug treatment, cells were exposed to CTX or STM2457 at predetermined working concentrations for the remainder of the culture period. Cell samples were harvested at designated time points dictated by the specific downstream assays.

### Cellular immunofluorescence

Cells were seeded on coverslips placed in 24-well plates at a density of 5.0  × 10^4^ cells per well. After culturing for 24 h prior to treatment, the cells underwent drug treatment and transfection. Following incubation, the cells were washed twice with PBS. They were then fixed with 4% paraformaldehyde (PFA) at room temperature for 20 min. After fixation, the cells were washed twice with PBS and permeabilized using PBS with 0.5% Triton X-100 at room temperature for 15 min. Subsequently, the cells were washed twice with PBS, blocked in PBST with 5% bovine serum albumin (BSA), and incubated overnight at 4 °C with primary antibodies against METTL3 (1:400, 15073-1-AP; Proteintech, Rosemont, IL, USA) and m6A (1:200, 68055-1-Ig; Proteintech, Rosemont, IL, USA). On the following day, cells were washed with PBS and incubated at room temperature for 90 min with species-specific secondary antibodies, followed by DAPI staining for 5 min. After a final wash with PBS, an anti-fade mounting medium was applied to prevent photobleaching and to mount the samples. Fluorescence signals were then visualized using a confocal microscope. Each experimental group included three biological replicates.

### Western blot and dot blot

Western Blot: KGN cells from different treatment groups were harvested, total protein was extracted, and protein concentration was measured using the bicinchoninic acid (BCA) assay (P0012; Beyotime, Shanghai, China). A total of 35 µg of protein per lane was resolved on a 10% SDS-PAGE gel and transferred to PVDF membranes at a constant voltage of 15 V for 45 min. After blocking with 5% non-fat milk at room temperature for 1 h, membranes were incubated overnight at 4 °C with primary antibodies against METTL3 (1:2000, 15073-1-AP; Proteintech, Rosemont, IL, USA) or GAPDH (1:2500, AC63048-100 µl; Acmec, Shanghai, China). The membranes were subsequently washed three times with TBST for 10 min, and then incubated at room temperature for 1 h with HRP-conjugated secondary antibodies (anti-rabbit IgG, 1:5000, Proteintech, SA00001-2; and anti-mouse IgG, 1:5000, SA00001-4; Proteintech, Rosemont, IL, USA). Following a final wash with TBST, protein bands were visualized using enhanced chemiluminescence (ECL). Each experimental group included three biological replicates.

Dot blot: Total RNA was adjusted to a concentration of 100 ng/µL, and aliquots containing 400 ng, 200 ng, and 100 ng were spotted onto Hybond-N+ membranes. The membranes were then dried and cross-linked under UV light for 5 min on each side. After washing twice with TBST (10 min per wash), the membranes were blocked with 5% non-fat milk for 1.5 h. Subsequently, the membranes were incubated overnight at 4 °C with m6A monoclonal mouse primary antibodies (1:2000, 68055-1-Ig; Proteintech, Rosemont, IL, USA). On the following day, after washing with TBST, HRP-conjugated secondary antibodies (1:5000, SA00001-4; Proteintech, Rosemont, IL, USA) were applied for 1 h at room temperature, followed by TBST washes and ECL detection. In parallel, a separate membrane served as a loading control and was spotted with RNA samples in the same manner. After UV cross-linking, it was stained with 0.1% methylene blue (MB) (G1300; Solarbio, Shanghai, China) for 5 min, and then rinsed with double-distilled water (ddH_2_O) to remove the excess MB. Each experimental group included three biological replicates.

### CCK-8 and EdU assays

KGN cells were resuspended at 5.0  × 10^4^ cells/mL. A total of 100 µL of cell suspension was seeded into a 96-well plate and cultured for 24 h before the cells were subjected to transfection or drug treatment. Subsequent assays were conducted 48 h post-transfection or drug treatment. For cell proliferation analysis, two assays were performed: the CCK-8 assay (PF00004; Proteintech, Rosemont, IL, USA) and the 5-ethynyl-2′-deoxyuridine (EdU) assay (C6045; UElandy, Suzhou, China). For the CCK-8 assay, 10 µL of CCK-8 reagent was added to each well. Following a 2 h incubation, absorbance at 450 nm (optical density (OD) value) was measured using a microplate reader. For the EdU assay, 10 µM EdU was added to each well, and the cells were incubated for 2 h. Subsequently, cells were fixed with 4% PFA at room temperature for 20 min. Residual PFA was then neutralized by incubating the cells with 2 mg/mL glycine solution at room temperature for 5 min, followed by permeabilization with 0.5% Triton X-100 at room temperature for 10 min. Following permeabilization, cells were washed twice with PBS supplemented with 3% BSA. Subsequently, cells were incubated with the EdU reaction mixture in the dark for 20 min. Cell nuclei were stained with DAPI. EdU-positive cells and total cell nuclei were visualized and imaged using a fluorescence microscope. Each experimental group included at least three biological replicates and three technical replicates.

### Mitochondrial membrane potential assay

Cells were seeded at densities of 5.0  × 10^5^ and 5.0  × 10^3^ cells per well in 6-well and 96-well plates, respectively, and pre-cultured for 24 h. After pre-culture, drug treatment and transfection were performed. JC-1 staining procedure was carried out according to the protocol of the JC-1 mitochondrial membrane potential detection kit (J6004; UElandy, Suzhou, China). For the positive control, cells were incubated with 50 µM carbonyl cyanide m-chlorophenylhydrazone (CCCP) at 37 °C for 20 min, followed by JC-1 staining. To stain the cells, an equal volume of JC-1 working solution was added to the cell culture medium in each well. The mixture was incubated at 37 °C for 20 min. After incubation, cells were washed with PBS and one mL of fresh culture medium was added. The red-to-green fluorescence ratio in the 6-well plates was assessed *via* fluorescence microscopy. For detection in the 96-well plate, after JC-1 staining, fluorescence signals were measured using a multimode microplate reader. Instrument parameters were set to measure excitation/emission wavelengths at 550/600 nm for red fluorescence and 485/535 nm for green fluorescence. Each experimental group included at least three biological replicates and three technical replicates.

### Statistics

Each experiment was independently repeated at least three times. Statistical analysis was performed using GraphPad Prism 8.0 software. Data are presented as mean ± SD. The independent samples *t*-test was used for comparisons between two groups. For multi-comparisons, one-way analysis of variance (ANOVA) was applied, followed by Dunnett’s multiple comparisons test for *post-hoc* analysis. A *P* < 0.05 value was considered statistically significant.

## Results

### Differentially expressed miRNAs identification and target genes prediction

**Figure 1:**
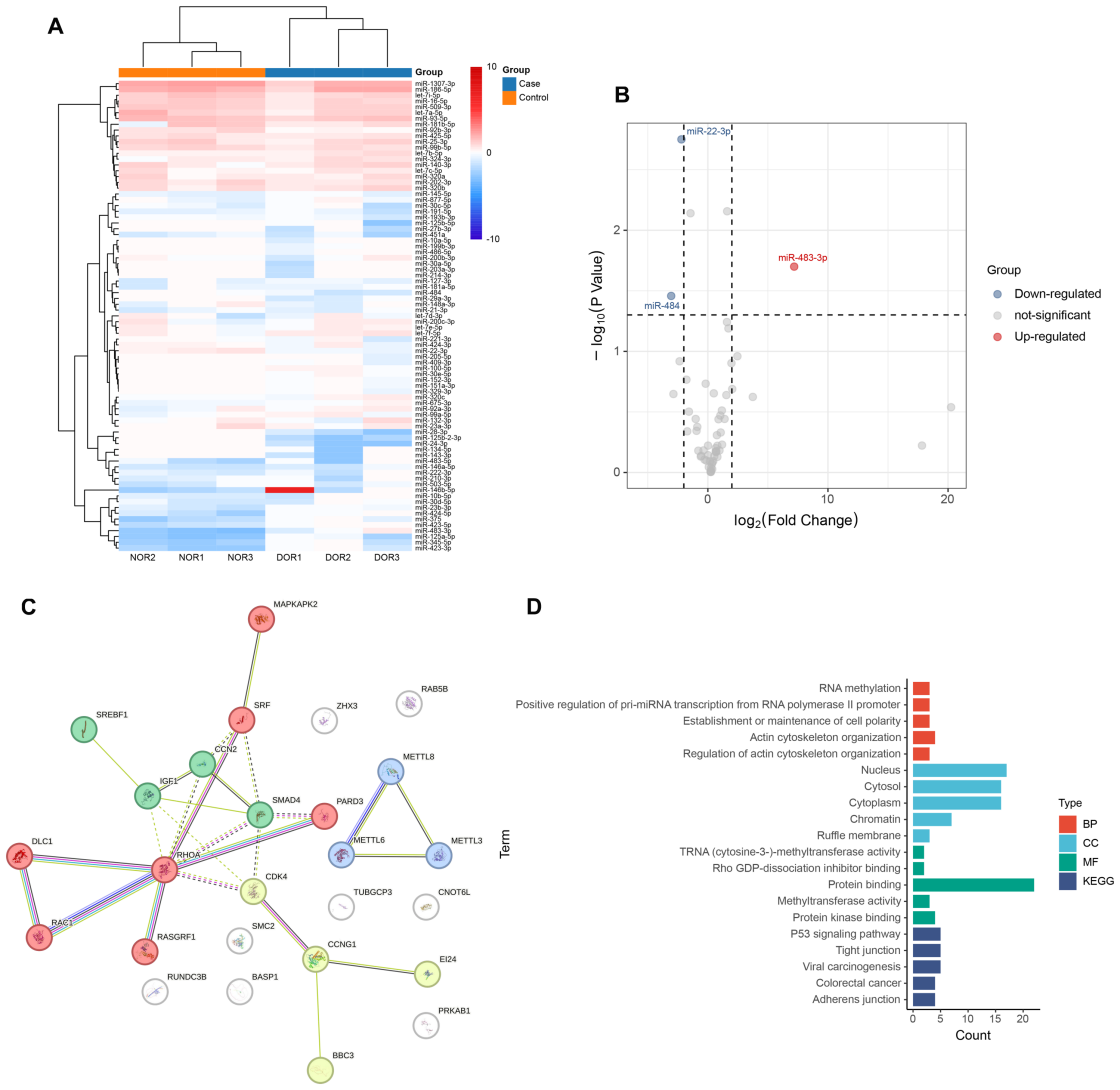
Differentially expressed miRNAs identification and target genes prediction. (A) Expression heatmap of the miRNA PCR array (red indicates high expression, blue indicates low expression). (B) Volcano plot of differentially expressed miRNAs. (C) Prediction of miR-483-3p target genes PPI network (Source: STRING database). (D) GO and KEGG analysis of the target genes predicted for miR-483-3p.

The results indicated that miR-483-3p was significantly upregulated in the exosomes of follicular fluid from patients with DOR, suggesting its potential role as a key regulatory molecule in the progression of DOR (Figs. 1A, 1B). Further, the PPI network of the target genes of miR-483-3p is shown in Fig. 1C. The PPI analysis revealed four main clusters, including *Apical junction assembly, p53 signaling pathway, FOXO-mediated transcription of oxidative stress, metabolic and neuronal genes, and tRNA (cytosine-3-)-methyltransferase activity* (shown in Data S1). It is noteworthy that the methyltransferases METTL3, METTL6, and METTL8 were identified as potential targets of miR-483-3p, indicating that this miRNA may regulate DOR progression by modulating RNA methylation (Fig. 1D).

### miR-483-3p directly targets METTL3 3′ UTR and suppresses its gene expression

**Figure 2:**
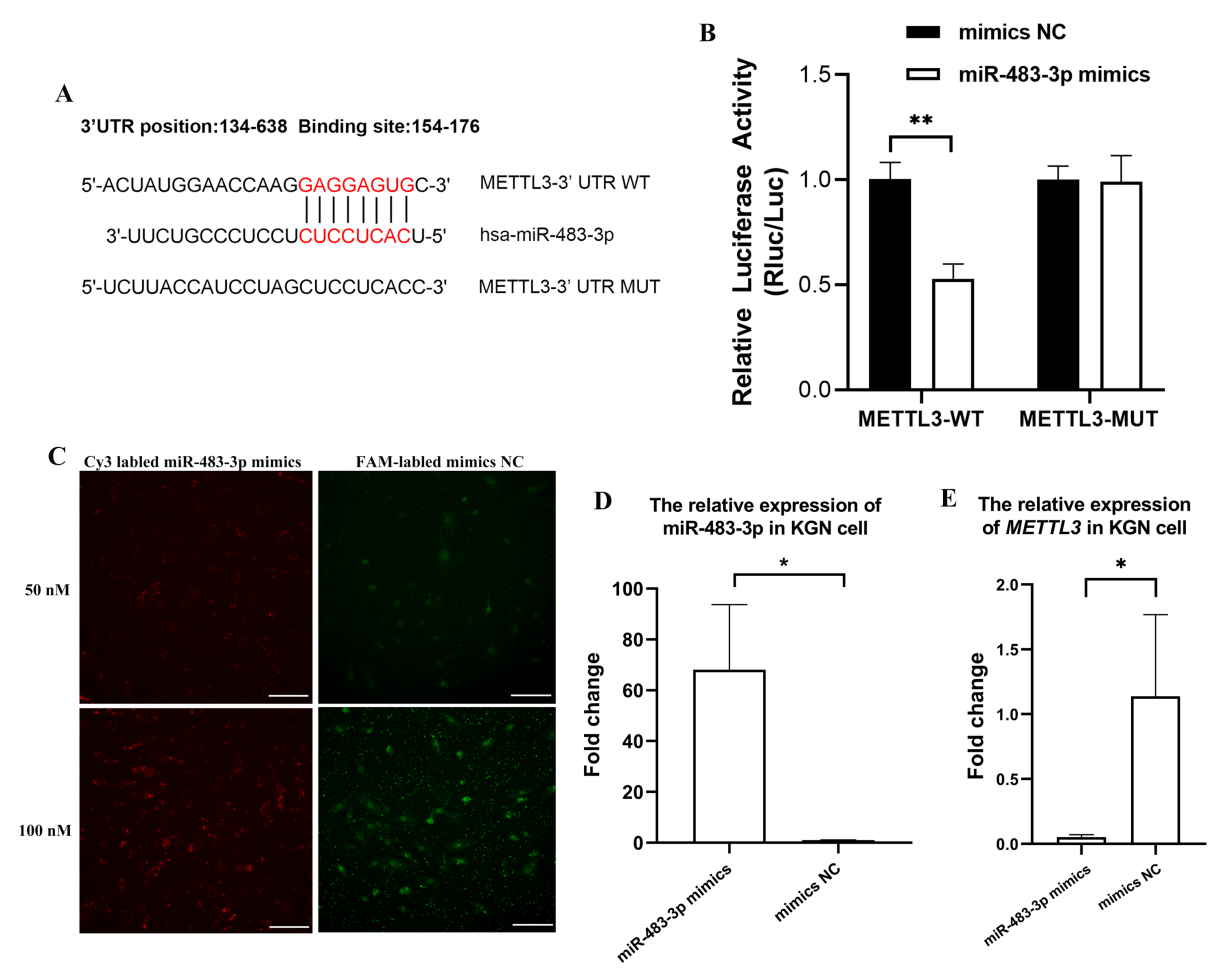
Dual-luciferase reporter gene assay and the effects of overexpression miR-483-3p on KGN cell. (A) Binding sites of miR-483-3p in the 3′ UTR of METTL3. (B) Dual-luciferase reporter gene assay. (C) Transfected miR-483-3p mimics or mimics NC into KGN cells (200 ×, bar = 100 µm). (D) The relative expression of miR-483-3p in KGN cells. (E) The relative expression of *METTL3* in KGN cells. *P < 0.05, * * *P* < 0.01.

Bioinformatics analysis revealed that he METTL3 3′ UTR contains specific binding sites for miR-483-3p (Fig. 2A). To investigate this, reporter plasmids containing either the wild-type METTL3 3′ UTR (METTL3-WT) or a mutant 3′ UTR (METTL3-MUT) were constructed. These plasmids, along with miR-483-3p mimics or mimics NC, were co-transfected into 293T cells. The results showed that luciferase activity was significantly lower in cells co-transfected with miR-483-3p mimics and METTL3-WT compared to those treated with mimics NC and METTL3-WT (*P* < 0.01; Fig. 2B). This finding indicates a direct interaction between miR-483-3p and the 3′ UTR of METTL3. To further validate this interaction, we investigated whether transfection of KGN cells with miR-483-3p mimics affects METTL3 expression. As shown in Fig. 2C, the transfection efficiency was optimal at 100 nM for both miR-483-3p mimics and mimics NC. After transfection, the miR-483-3p expression significantly increased, whereas the METTL3 expression markedly decreased (*P* < 0.05; Figs. 2D, 2E).

### Effects of different treatments on METTL3 expression and m6A levels in KGN cells

**Figure 3:**
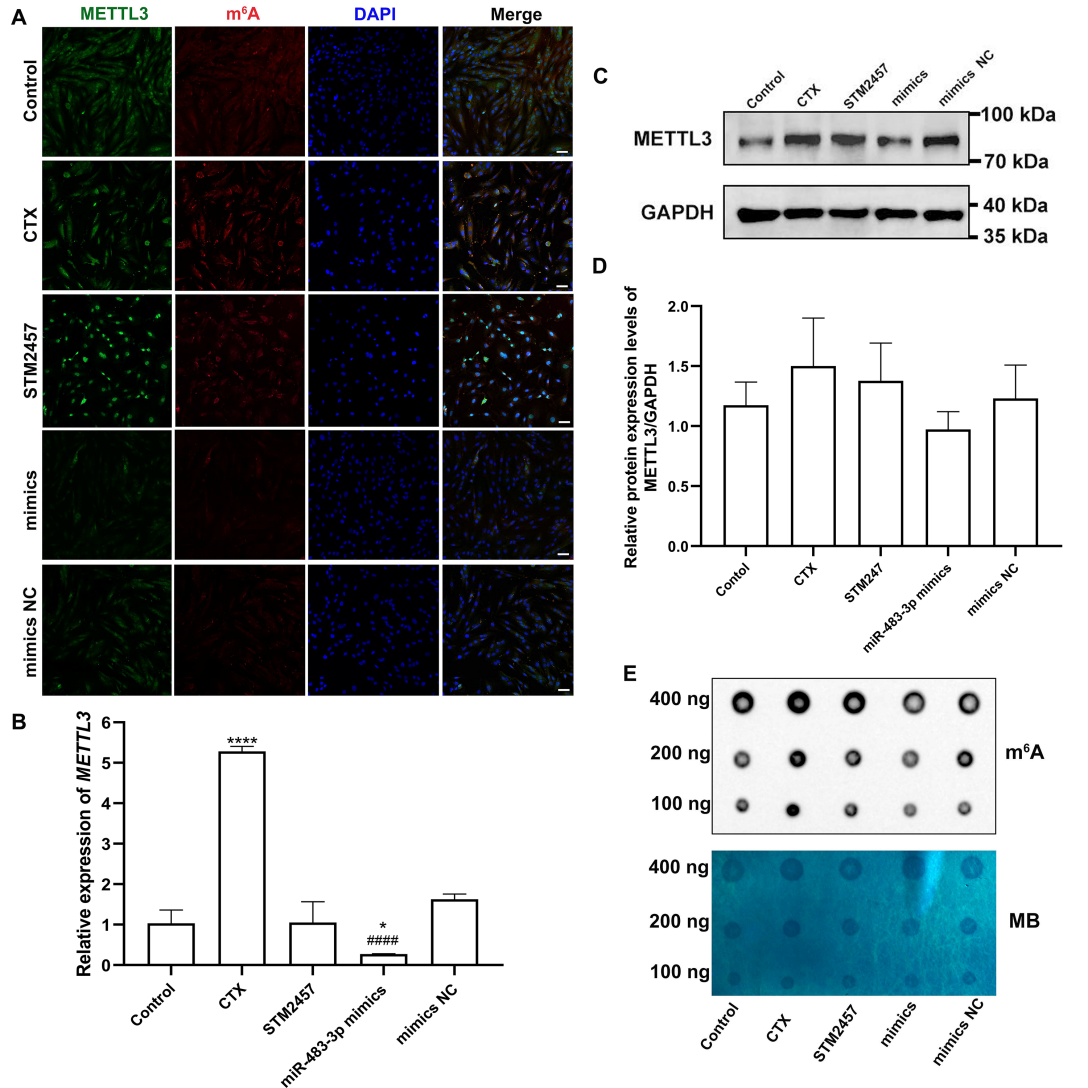
The effects of different treatments on the METTL3 mRNA expression, protein and m6A levels in KGN cells. (A) The immunofluorescence signals of KGN cells in different groups (200 ×, bar = 50 µm). (B) The relative METTL3 mRNA expression in different groups. (C) Western blot assay of METTL3 in different groups. (D) The relative protein expression levels of METTL3 in different groups. (E) Dot blot assay of m6A levels in different groups. Compared to the control group, * *P* < 0.05, **** *P* < 0.0001. mimics group *vs.* mimics NC group, ## *P* < 0.01.

The immunofluorescence assay revealed a decrease in METTL3 protein fluorescence intensity in the miR-483-3p mimics group. In contrast, m6A levels were highest in the CTX-treated group (Fig. 3A). Results showed that miR-483-3p mimics significantly suppressed METTL3 mRNA expression compared to mimics NC (*P* < 0.0001; Fig. 3B). In addition, the CTX treatment markedly increased METTL3 expression in KGN cells relative to the control group (*P* < 0.0001; Fig. 3B), whereas miR-483-3p mimics produced an opposite effect. Western blot and Dot blot assays further confirmed a similar increasing trend in METTL3 protein expression and m6A levels in the CTX group (Figs. 3C, 3D and 3E). Unexpectedly, both METTL3 protein and m6A levels increased after STM2457 treatment, paralleling the trend observed in the CTX-treated group (Figs. 3A, 3C, 3D and Fig. 3E). Notably, although miR-483-3p mimics significantly suppressed METTL3 mRNA levels, the differences in METTL3 protein and m6A levels among the control, miR-483-3p mimics, and mimics NC groups were comparatively modest.

### Effects of different treatments on the proliferative capacity and mitochondrial function of KGN cells

**Figure 4:**
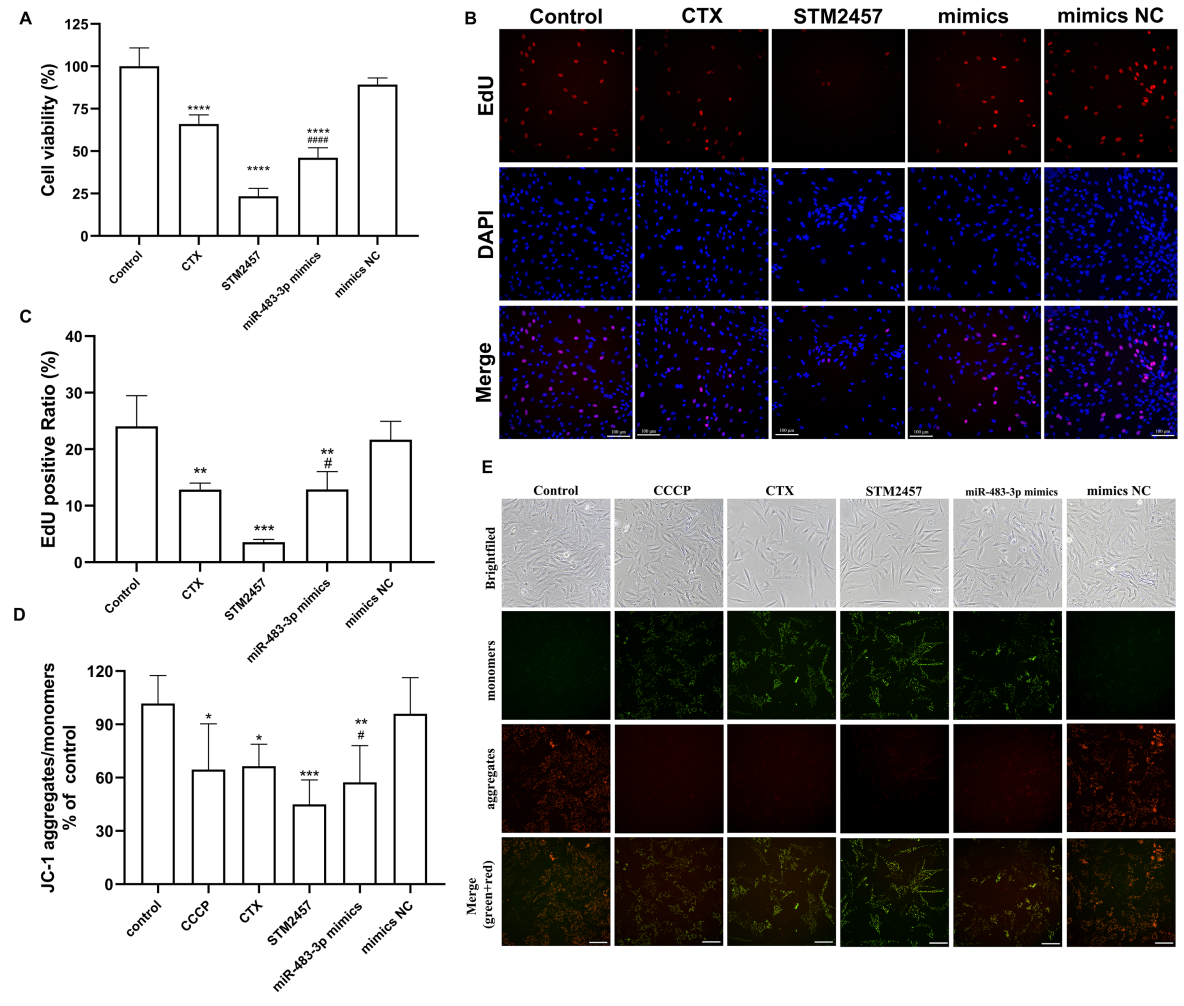
The effects of different treatments on the proliferation and MMP in KGN cells. (A) The cell viability of KGN cells was analyzed by CCK-8 array. (B) The EdU staining of KGN cells in different groups (200 ×, bar = 100 µm). (C) The EdU positive ratio of KGN cells. (D) The JC-1 aggregates/monomers ratio in different groups (The control group normalized as 1). (E) The fluorescence signals of JC-1 aggregates and monomers in different groups (200 ×, bar = 100 µm). Compared to control group, * *P* < 0.05, ** *P* < 0.01, *** *P* < 0.001, **** *P* < 0.0001. mimics group *vs.* mimics NC, # *P* < 0.05, #### *P* < 0.0001.

Compared to the control group, CTX, STM2457, and miR-483-3p mimics significantly reduced the KGN cell viability (*P* < 0.0001; Fig. 4A). Among them, STM2457 exhibited the strongest inhibitory effect. Furthermore, EdU assays revealed that treatments with CTX, STM2457, and miR-483-3p mimics resulted in a notable decrease in the proportion of EdU-positive cells, with the STM2457 group showing the most significant reduction (*P* < 0.01; Figs. 4B, 4C). To further assess mitochondrial function, JC-1 staining assays indicated that, compared to the control group, treatments with the mitochondrial uncoupler CCCP, CTX, STM2457, and miR-483-3p mimics all significantly reduced the JC-1 red to green fluorescence intensity ratio, which reflects the level of mitochondrial membrane potential. Notably, the STM2457 group exhibited the most pronounced reduction in mitochondrial membrane potential in KGN cells (*P* < 0.001; Figs. 4D, 4E).

## Discussion

DOR is a significant reproductive health issue primarily characterized by a decline in ovarian reserve, which ultimately compromises female fertility. Clinically, it manifests as irregular menstrual cycles, hormonal dysregulation, and infertility (Bochynska et al., 2025). The pathogenesis of DOR is complex and heterogeneous, attributed to a multifactorial etiology encompassing genetic predisposition, autoimmune responses, metabolic syndrome, and infectious agents (Robeva et al., 2024; Tian et al., 2025; Zhang et al., 2024). However, the molecular basis underlying DOR remains incompletely elucidated. In particular, the regulatory mechanisms of certain key signaling pathways require further investigation.

Follicular fluid (FF) is a crucial component of the follicular microenvironment. It provides essential support for the growth and development of various follicular cells and facilitates intercellular communication (Wang et al., 2024). As key regulators within FF, follicular fluid exosomes influence follicular development, oocyte maturation, and related signaling pathways (Tesfaye, Menjivar & Gebremedhn, 2021). Studies have shown that dynamic alterations in the miRNA expression profile of follicular fluid exosomes are closely associated with the developmental status of follicles and oocytes (Zhang et al., 2021; Zhou et al., 2024), and aberrant expression of these miRNAs can lead to ovarian dysfunction and the occurrence of related complications (Cao et al., 2022; Zhou et al., 2022). Our study revealed that the expression of miR-483-3p was detected in follicular fluid-derived exosomes from both DOR and normal patients, but was significantly higher in DOR samples. We hypothesized that in DOR patients, disruption of the follicular microenvironment may further upregulate the expression of exosomal miR-483-3p, thereby inducing dysregulation of downstream gene networks. This cascade could potentially contribute to the persistence or aggravation of the DOR phenotype.miR-483-3p, acting both as a regulator and a biomarker in various diseases, has been shown to influence the cell cycle and apoptosis pathways by targeting insulin-like growth factor-1 (IGF-1) in tumors and metabolic diseases, thereby affecting cell fate (Wang et al., 2020). Moreover, exosomal miR-483-3p modulates immune responses induced by viral infections, particularly antiviral cellular immune responses (Maemura, Fukuyama & Kawaoka, 2020; Maemura et al., 2018) and has been validated as an important liquid biopsy biomarker for disease progression in conditions such as sepsis and pancreatic ductal adenocarcinoma (Qiu et al., 2022; Shao et al., 2021). Evidence indicates that miR-483-3p can induce ovarian dysfunction by targeting and suppressing hormone receptors or steroid synthesis-related genes in GCs (Rogers et al., 2025). Clinical studies further demonstrate that elevated miR-483-3p expression in GCs is often associated with a significant decline in oocyte quality and developmental potential (Liu et al., 2022b). Our findings demonstrate that miR-483-3p directly targets METTL3. Other studies have demonstrated that abnormal m6A modification mediated by METTL3 can lead to manifestations of ovarian hypofunction, such as reduced ovarian reserve, ovarian cell aging, and impaired follicular development (Huang et al., 2019; Mu et al., 2021). This suggests that the accumulation of exosome-derived miR-483-3p in GCs may contribute to the pathological progression of DOR by interfering with METTL3-mediated m6A modification and its downstream signaling pathways.

This study aimed to elucidate the effects of miR-483-3p overexpression on GCs. Specifically, KGN cells were treated individually with either CTX, an agent commonly used to induce animal models of premature ovarian failure (POF) and DOR, or STM2457, a highly potent and selective inhibitor of METTL3 catalytic activity. CTX promotes granulosa cell death by inducing mitochondrial dysfunction, increasing reactive oxygen species (ROS) levels, and triggering ferroptosis, ultimately leading to ovarian dysfunction (Chen et al., 2024b; Zhang et al., 2023). STM2457 inhibits the growth of acute myeloid leukemia (AML) cells while promoting their differentiation and apoptosis *via* suppressing METTL3 enzymatic activity (Yankova et al., 2021). Furthermore, studies have demonstrated that STM2457 can reduce drug resistance in hepatocellular carcinoma cells, a process promoted by METTL3-mediated m6A (Wang et al., 2023). The effects of miR-483-3p overexpression were compared against those of these treatments on GCs to elucidate their relative impacts. Our *in vitro* experiments revealed that miR-483-3p overexpression, CTX, and STM2457 all significantly suppressed the proliferation of KGN cells to a comparable extent. CTX is a prodrug that requires hepatic activation *via* CYP450 enzymes to generate its active metabolite, 4-hydroxycyclophosphamide/phosphoramide mustard. Conventionally, effective injury models are established by co-incubating metabolic enzymes with the parent compound or by utilizing CTX derivatives. To align with the 48-hour transfection window for miR-483-3p, this study adopted the experimental approach described by Liu et al. (2022a) who reported that prolonged, high-concentration (250 µM, 48 h) *in vitro* exposure to CTX successfully established a CTX-induced KGN cell injury model. Consistently, we observed that treatment with CTX (500 µM, 48 h) also effectively established a KGN cell injury model. Notably, although this study did not experimentally assess apoptosis or cell cycle progression in CTX- and STM2457-treated KGN cells, morphological examination indicated that these cells exhibited characteristic apoptotic features. These observations suggest that overexpression of miR-483-3p may promote apoptosis by disrupting METTL3 function, a mechanism that merits further exploration in future studies.

Moreover, a comparative analysis of experimental data from various treatment groups revealed a significant upregulation of METTL3 expression and m6A levels in the CTX group. A study found that CTX treatment of KGN cells and mice significantly increased the levels of METTL3 protein and m6A modification (Huang et al., 2019). This suggests that the mechanism of ovarian damage induced by miR-483-3p inhibition of METTL3 differs significantly from that induced by CTX. As expected, our experimental results showed that STM2457 did not decrease METTL3 mRNA expression. Unexpectedly, both METTL3 protein and m6A levels were elevated after STM2457 treatment, paralleling the trend observed in the CTX-treated group. Previous studies have reported that higher METTL3 expression and METTL3-mediated m6A levels significantly decreased cell viability and promoted apoptosis (Chen et al., 2024a; Liao et al., 2025). These findings suggest that the cytotoxic effects of STM2457 may enhance METTL3-mediated m6A modification activity, thereby further exacerbating apoptosis. The above findings indicate that under normal physiological conditions, miR-483-3p may play a role in maintaining normal ovarian function. However, its excessive accumulation may induce damage to GCs, leading to impaired ovarian function. This mechanism differs from the toxic injury induced by CTX, STM2457, or other drugs. Although this study is limited by the semi-quantitative nature of Dot blot and Western blot assays—which confined our analysis to global m6A levels—and by the marked heterogeneity in METTL3 expression profiles, the miR-483-3p-METTL3-m6A axis remains a compelling target for future research into ovarian function. Implementing more precise quantitative techniques will be key to fully deciphering this regulatory network.

The results of this study not only provide experimental evidence for exploring the mechanism of drug-induced ovarian damage but also offer a basis for proposing new intervention strategies. Reducing the excessive accumulation of small non-coding RNAs, such as miR-483-3p, in ovarian tissues could mitigate damage to ovarian cells and restore ovarian function.

### Limitations of the study

The study has several limitations that should be considered: (1) Despite strict adherence to DOR criteria for patient specimen screening, with AMH levels as a mandatory reference, the limited sample size precluded statistically significant differences in clinical characteristics between groups. This may affect the reliability of miRNA screening and should be carefully considered in further assessments. (2) The absence of prospective clinical validation means that the current results need further confirmation through real-world clinical studies. (3) Reliance primarily on *in vitro* models limits a comprehensive understanding of the specific regulatory functions of miR-483-3p under both normal physiological and pathological conditions *in vivo*. (4) Variability between experimental batches, encompassing technical and biological replicates across different datasets, may amplify overall variability and introduce bias, potentially compromising the reliability of the outcomes. To address these limitations, future studies should prioritize expanding the sample size and incorporating rigorous clinical validation. Therefore, constructing *in vivo* functional experimental models would enhance the scientific rigor and practical applicability of the results.

## Conclusions

In conclusion, this study provides preliminary evidence suggesting a specific regulatory interaction between miR-483-3p and METTL3. We demonstrate that aberrantly high expression of miR-483-3p significantly suppresses METTL3 mRNA levels but only moderately reduces METTL3 protein and m6A levels, suggesting the involvement of complex post-transcriptional regulatory mechanisms. Furthermore, overexpression of miR-483-3p markedly inhibits the proliferation of ovarian GCs and impairs their mitochondrial function. Further investigation into the role of the candidate miR-483-3p-METTL3-m6A axis in DOR will be conducted using *in vivo* models alongside an expanded cohort of clinical samples.

##  Supplemental Information

10.7717/peerj.21567/supp-1 Supplemental Information 1 The raw data of miRNA PCR array

10.7717/peerj.21567/supp-2 Supplemental Information 2 Raw data of qRT-PCR

10.7717/peerj.21567/supp-3 Supplemental Information 3 Raw data of Dual-luciferase report gene assay

10.7717/peerj.21567/supp-4 Supplemental Information 4 Raw data of EdU

10.7717/peerj.21567/supp-5 Supplemental Information 5 Raw data of JC-1

10.7717/peerj.21567/supp-6 Supplemental Information 6 Raw data of CCK8

10.7717/peerj.21567/supp-7 Supplemental Information 7 Raw data of WB

10.7717/peerj.21567/supp-8 Supplemental Information 8 Raw data of DOT blot

10.7717/peerj.21567/supp-9 Supplemental Information 9 MIQE checklist

10.7717/peerj.21567/supp-10 Supplemental Information 10 Drug concentration screening of CTX and STM2457 (A) The cell viability of KGN cell treated by different CTX concentration. (B) The cell viability of KGN cell treated by different STM2457 concentration. Compared to control group, *P ¡0.05, *** P ¡0.001, **** P ¡0.0001.

## Additional Information and Declarations

### Data Availability

The following information was supplied regarding data availability: The data is available in the Supplemental Files.
