# Supplementary material for: Overaccumulation of miR-483-3p exerts acute toxic effects on ovarian granulosa cells by impairing cell proliferation, mitochondrial function, and METTL3-mediated m6A modification
Source: PeerJ. 2026 Jul 27;14:e21567. doi: 10.7717/peerj.21567 (PMC13421807; doi:10.7717/peerj.21567)
Supplement: Supplemental Information 10 — (A) The cell viability of KGN cell treated by different CTX concentration. (B) The cell viability of KGN cell treated by different STM2457 concentration. Compared to control group, *P ¡0.05, *** P ¡0.001, **** P ¡0.0001. [file peerj-14-21567-s010.pdf]

A

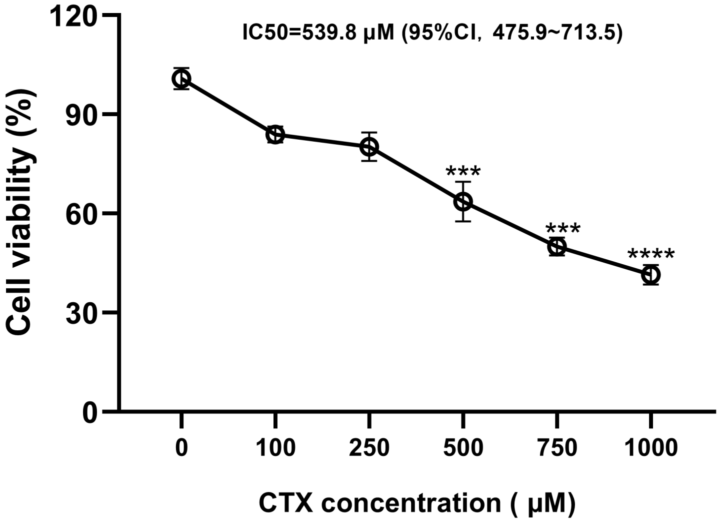

B

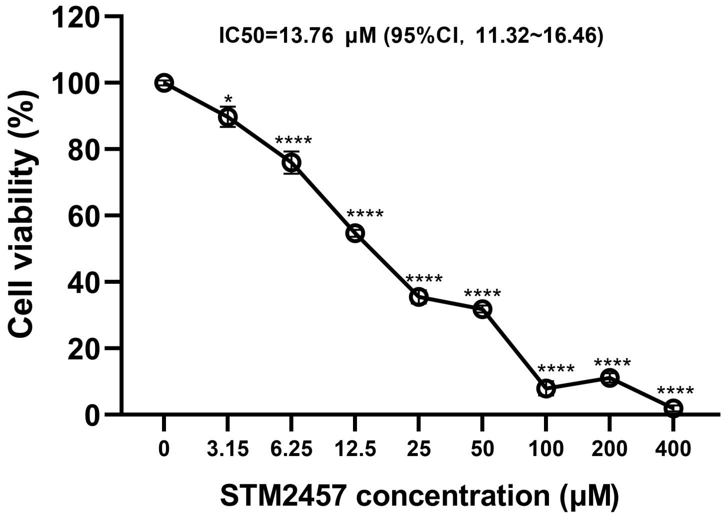

Figures S1 Drug concentration screening of CTX and STM2457. (A) The cell viability of KGN cell treated by different CTX concentration. (B) The cell viability of KGN cell treated by different STM2457 concentration. Compared to control group, *P<0.05, *** P<0.001， **** P<0.0001.

Legend: KGN cells were treated with different drug concentrations for 48 h, and cytotoxicity was evaluated using the CCK-8 assay. The results showed that, compared with the control group, when the CTX concentration reached 500 μM, cell viability significantly decreased (P < 0.001), with an IC_{50} value of 539.8 μM (95% CI: 475.9–713.5). Furthermore, when the STM2457 concentration reached 3.15 μM, cell viability also significantly decreased (P < 0.05), with an IC_{50} value of 13.76 μM (95% CI: 11.32–16.46). Therefore, this study selected CTX at 500 μM and STM2457 at 12.5 μM, which were administered for 48 h, as the experimental conditions to induce damage in KGN cells.
